# Secondary Membranous Nephropathy. A Narrative Review

**DOI:** 10.3389/fmed.2020.611317

**Published:** 2020-12-03

**Authors:** Gabriella Moroni, Claudio Ponticelli

**Affiliations:** ^1^Nephrology Unit Fondazione Istituto di Ricovero e Cura a Carattere Scientifico (IRCCS) Ca' Granda Ospedale Maggiore, Milan, Italy; ^2^Retired, Milan, Italy

**Keywords:** NSAIDs, HBV infections, cancer, membranous lupus nephropathy, secondary membranous nephropathy, primary membranous nephropathy

## Abstract

Membranous nephropathy (MN) is a common cause of proteinuria and nephrotic syndrome all over the world. It can be subdivided into primary and secondary forms. Primary form is an autoimmune disease clinically characterized by nephrotic syndrome and slow progression. It accounts for ~70% cases of MN. In the remaining cases MN may be secondary to well-defined causes, including infections, drugs, cancer, or autoimmune diseases, such as systemic lupus erythematosus (SLE), rheumatoid arthritis (RA), urticarial vasculitis, sarcoidosis, thyroiditis, Sjogren syndrome, systemic sclerosis, or ankylosing spondylitis. The clinical presentation is similar in primary and secondary MN. However, the outcome may be different, being often related to that of the original disease in secondary MN. Also, the treatment may be different, being targeted to the etiologic cause in secondary MN. Thus, the differential diagnosis between primary and secondary MN is critical and should be based not only on history and clinical features of the patient but also on immunofluorescence and electron microscopy analysis of renal biopsy as well as on the research of circulating antibodies. The identification of the pathologic events underlying a secondary MN is of paramount importance, since the eradication of the etiologic factors may be followed by remission or definitive cure of MN. In this review we report the main diseases and drugs responsible of secondary MN, the outcome and the pathogenesis of renal disease in different settings and the possible treatments.

## Introduction

**Figure 1:**
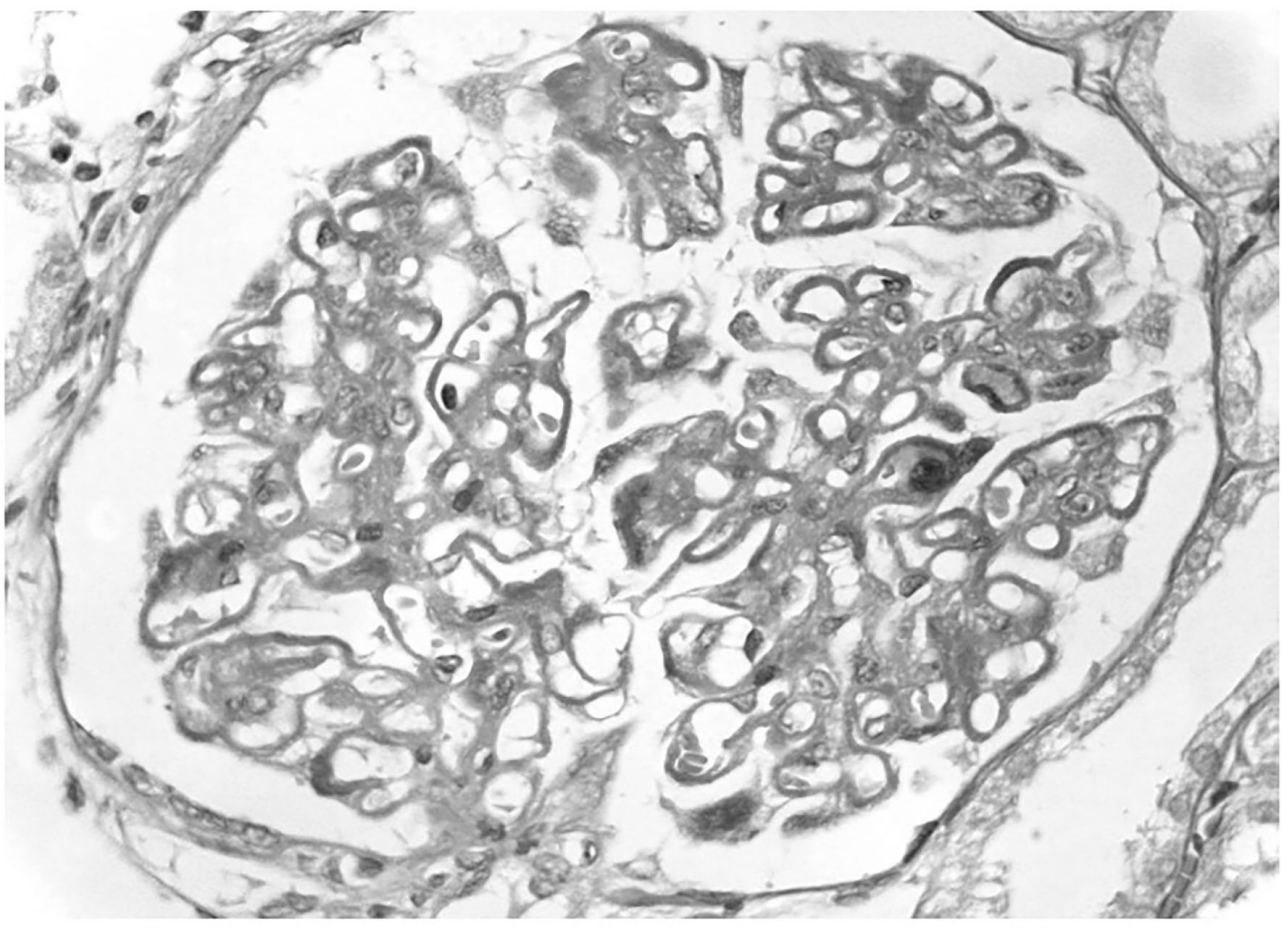
Primary membranous nephropathy. A light microscopy there is diffuse thickening of glomerular capillary walls due to the presence of many immunedeposits in subepithelial position.

The term membranous nephropathy (MN) indicates a pathological condition characterized, at light microscopy, by thickening of the glomerular basement membrane (GBM), which is diffuse to all glomeruli and involves the whole glomerulus. In most cases MN is an autoimmune disease caused by autoantibodies directed against phospholipase A2 receptor (PLA2R) or, more rarely, thrombospondin type-1 domain-containing 7A (THSD7A) (1, 2). However, the antigen THSD7A is not specific for primary MN; it can also be detected in MN patients with cancer. When secondary causes are excluded, the disease is called primary MN (Figure 1). Immunofluorescence analysis shows granular sub-epithelial deposits of immunoglobulin G (mainly IgG4) and C3, with lesser amounts of IgM or IgA and uncommonly C1q, suggesting that there is not complement activation by the classical pathway (3). Electron microscopy can detect deposits of varying electron density and shape confined to the subepithelial space of glomeruli or incorporated into irregular projections of GBM-like material (“spikes and domes”). Mesangial electron deposits are absent or scanty in primary MN (Table 1).

**Table 1:** Differential diagnosis from primary to secondary membranous nephropathy at renal biopsy.

	**Primary membranous nephropathy**	**Secondary membranous nephropathy**
Light microscopy	Uniform thickening of GBM diffuse to all glomeruli. No proliferation.	Endocapillary hypercellularity may be seen in MN secondary to SLE or cancer. Mesangial proliferation in MN secondary to SLE, cancer, or Sjogren syndrome.
Immuofluorescence	Subepithelial deposits of IgG (usually IgG4) and C3. Staining with PLA2R (70% of cases).	Subepithelial deposits of IgG (usually IgG1, IgG2, or -IgG3), C1q, IgA, IgM in SLE, cancer, and in some cases of drug-induced MN. Staining with PLA2R in some cases of HBV infection, S.mansoni, SLE, cancer, sarcoidosis.
Electron microscopy	Subepithelial electron-dense deposits.	Subepithelial and subendothelial deposits in HBV, SLE, Tubulo-interstitium and vessels deposits in SLE.

However, in some cases a picture of MN at light microscopy may be associated with infection, drug exposure, cancer, or other autoimmune diseases (Table 1). These secondary MNs may show peculiar aspects at immunofluorescence and electron microscopy and may have different clinical presentation and natural course. Any effort should be made to distinguish primary from secondary MN, since their treatment may be different and sometimes opposed with devastating consequences in case of wrong.

A narrative review was performed to identify cases of MN associated with different types of infections or developed during or after the use of drugs, or secondary to malignancy, or autoimmune diseases. We reviewed the literature by searching for the following terms on Pubmed.gov: Membranous nephropathy, Secondary Membranous Nephropathy, Infection and Glomerulonephritis, Drugs and Glomerulonephritis, Autoimmune disease and Glomerulonephritis, Cancer and Glomerulonephritis, Lupus membranous nephropathy, Nephrotic Syndrome, Rheumatoid Arthritis, Sarcoidosis, IgG4 disease, Urticarial vasculitis, Hematopoietic Stem Cell Transplant, Thyroiditis, Systemic Sclerosis, Sjogren Syndrome, Ankylosing spondylitis. We selected the papers reporting cases of secondary membranous nephropathy.

## Post-Infective Membranous Nephropathy

### Viral Infections

Membranous nephropathy is the most common extrahepatic manifestation of hepatitis B virus (HBV) infection. It is generally associated with active viral replication, as indicated by the presence of B-viral DNA and hepatitis B antigen. At time of diagnosis of MN, liver enzymes may be normal or only mildly elevated. The prevalence of HBV-associated MN is strictly correlated with the geographic prevalence of HBV infections. In recent years, the use of HBV vaccine allowed to minimize the diffusion of HBV infection in developed countries. The few cases, reported nowadays in the Western areas, occur in high-risk subjects such as in intravenous drug addicts (4), but in tropical countries HBV-associated MN remains a frequent cause of nephrotic syndrome, particularly in children.

The light microscopic histological appearances are similar to idiopathic MN, but mild mesangial proliferation may be seen in HBV-MN; on electron microscopy there are typical subepithelial deposits and a few subendothelial deposits. The demonstration by immunofluorescence of the presence HBV antigens, mainly HBe, in form of granular deposits along the GBM, may support the pathogenetic role of HBV infections in the development of glomerular diseases (5). Theoretically, the small size and the cationic charge of this molecular weight antigen might pass through the GBM and localize in the subepithelial area eliciting the formation of antibodies (6). However, there is currently little evidence to support this hypothesis. In a Chinese study, 25 of 39 (64%) patients with HBV-associated MN showed PLA2R overlapped with HBsAg along the capillary loop, suggesting that HBV antigen may colocalize with anti-PLA2R antibody (7). As in idiopathic forms, the clinical presentation of HBV-related MN is characterized both in children and in adults, by variable degree of proteinuria from mild to nephrotic extent, often associated with microscopic hematuria. Hypocomplementemia has been reported in some cases in the initial phases of the disease. The clinical course seems to be different in children and in adults. The diagnosis of MN in children was generally done during a screening campaign. Most children with HBV- associated MN had a spontaneous remission of proteinuria within 1 year after the diagnosis. In a minority of children proteinuria persisted and chronic renal insufficiency or end stage renal failure (ESRD) developed later (8). In adults, spontaneous remission of proteinuria is infrequent, and the clinical course is more frequently progressive. In a cohort of 21 adults with HBV related MN, after a mean follow-up of 60 months, 29% developed chronic renal insufficiency and 10% entered ESRD (9). In patients with abnormal liver function tests and nephrotic syndrome the progression to ESRD was more rapid (10).

Few cases of MN have been diagnosed in HCV-positive patients (11). The clinical presentation is characterized by nephrotic proteinuria. Hypocomplementemia have been reported rarely, while cryoglobulin and rheumatoid factors were absent. The demonstration by indirect immunofluorescence of presence of HCV core RNA in the glomerular deposits in two patients with MN supports the role of HCV infections in the pathogenesis of these forms of MN (12). Glucocorticoids or rituximab in both hepatitis B/C viral-associated MN resulted ineffective and contraindicated as these drugs can increase viral replication. However, rituximab may obtain reduction of HCV in cryoglobulinemic glomerulonephritis (13) Antiviral therapy may obtain remission of MN (14). Tenofovir, entecavir, telbivudine, and lamivudine may obtain HBeAg clearance and remission of proteinuria (15, 16). Tacrolimus combined with entecavir rapidly and effectively induced partial or complete remission of HBV-MN in a series of Chinese adults (17).

Cases of MN in patients affected by HIV infection have also been reported (18). It is likely that other viruses, including influenza vaccination (19), may be involved in the pathogenesis of secondary MN. The diagnostic criteria for virus-related nephropathy include detailed clinical and laboratory data, and tissue molecular analysis. Several mechanisms are involved in the pathogenesis of virus-related nephropathy, including tropism of the virus in the kidney, induction of abnormal immune complexes, direct cytopathogenic effects, and multiorgan failure (20).

### Parasitic and Bacterial Infections

Schistosomiasis is a parasitic disease caused by organisms from the genus Schistosoma. The incidence of glomerular involvement in the various forms of schistosomiasis is estimated in 5–6% and increases to 15% in the hepatosplenic form (21). The association of MN with schistosomiasis is infrequent (22, 23). In old studies, antigens from Schistosoma mansoni have been found in the sera of humans and animals infected with the parasite, suggesting that MN was a secondary form (24, 25). However, recent reports challenged this interpretation. The analysis of renal biopsy demonstrated that at light microscopy there was thickening of the GBM often associated with granulomatous reaction in the renal interstitium. Immunofluorescence revealed granular deposits of IgG1 and IgG4 and electron microscopy indicated subepithelial electron-dense deposits. Surprisingly, a diffuse staining with PLA2R antibodies was observed (26, 27), suggesting the possibility of the coincidental presence of schistosomiasis and MN. The few available studies reported that MN was refractory both to the standard treatment used for primary MN and to specific antiparasitic treatment with praziquantel alone or in combination with artemether or artesunate.

In Plasmodium malaria, Filariasis and Mycobacterium leprosy (28, 29) membranoproliferative and mesangioproliferative glomerulonephritis are prevalent, while MN is infrequent.

Infection of syphilis can also be associated with MN. Treatment with penicillin for secondary syphilis can obtain normalization of renal function and resolution of the nephrotic syndrome (30).

## Drug Induced Membranous Nephropathy

Membranous nephropathy secondary to drug exposure is not infrequent. In a single center experience, in out of 129 patients with MN, an underlying cause was identified in 40 cases (31%). In 18 of them (45%) MN was secondary to drugs (31).

The pathogenetic mechanism of drug-induced MN is probably due to an immune response to the drug or to a by-product that acts as planted antigen on the subepithelial position of the GBM. The most plausible mechanism is that cationic drug–derived antigens traverse the GBM, are planted in the subepithelial space, and are targeted *in situ* by circulating antibodies directed against these antigens (32), leading to alterations of the GBM and glomerular filtration barrier eventually resulting in proteinuria. Patients with drug-induced nephrotic syndrome frequently have the HLA-B8 and DR3 antigens (33).

At renal biopsy, drug-induced MN is not different from primary forms. In the past, the most frequent drugs that caused MN were gold salts, penicillamine, and bucillamine that contained a sulfhydryl group, also called Thiol group. However, the use of these drugs has progressively reduced after the introduction of biological agents in the treatment of rheumatoid arthritis. Nevertheless, cases of MN secondary to the use of the monoclonal antibody adalimumab (34). A patient with rheumatoid arthritis and osteoporosis developed MN after treatment with denosumab (35). Some cases of MN have been reported with the use of Captopril. The development of MN can be attributed to a sulfhydryl group which is present in captopril but is absent in other ACE inhibitors (36). As a matter of fact, no other drugs of ACE family have been reported to induce MN. A rare case of lithium associated MN has been reported in an adolescent (37). Another rare cause of MN was chronic mercury exposure secondary to occupational exposures, contaminated fish, dental amalgams, but also cosmetics such as skin-lightening creams (38). Eleven patients were described by Li et al., all had normal function at presentation and proteinuria was in nephrotic range in 3 cases only. At light microscopy mild mesangial proliferation, and some leukocytes were present in the capillary lumen. At immunofluorescence IgG1 was predominant with C3, but other immunoglobulins and C1q were present (39). In most cases of drug-induced MN the disease remitted after withdrawal of the offending drug, sometimes years later.

Membranous nephropathy may also develop during the exposure to non-steroidal anti-inflammatory drugs (NSAIDs). MN was reported with all NSAIDs including selective cyclooxygenase-2 inhibitors, suggesting that the possible mechanism of action on renal damage could be mediated through their common action on cyclooxygenase inhibition (40). The exact rate of MN in patients in treatment with NSAIDs is not known. A retrospective study at the Mayo Clinic reported that 13 out of 125 cases (10.4%) of MN stage I or II diagnosed between 1975 and 1995 met the criteria for NSAIDs-associated MN (41). Patients with NSAIDs associated MN were generally older than those with idiopathic forms, probably due to the widely use of these drugs in old people. The duration of NSAIDs treatment before the development of MN is extremely variable from few weeks too years. Another characteristic is the very rapid development of nephrotic proteinuria, the hallmark of the disease. This rapid development of proteinuria explains the early stage of MN (class I or II) at renal biopsy. In most cases the withdrawal of the offending drug allows the remission of proteinuria without the need of immunosuppressive therapy. However, proteinuria may take some months before disappearing. Immune deposits can also resolve completely at repeated renal biopsy (42). The disease does not recur even after a long-term follow-up.

## Cow's Milk

A particular and rare form of secondary MN is caused by cow's milk. High levels of cationic circulating anti-bovine serum albumin (BSA) antibodies of IgG1 and IgG4 subclasses may be detected in these cases, and BSA may be recognized in subepithelial immune deposits. These data suggest that in a few children with MN, cationic BSA introduced with cow's milk may result in pathogenic MN if it passes the intestinal barrier. Once in the blood, BSA may bind to the anionic glomerular capillary wall, be reached by antibodies, and cause *in situ* formation of immune complexes (43). However, it is still unknown if removing cow's milk from the diet of an affected patient with anti–bovine serum albumin antibodies can modulate MN (44).

## Membranous Nephropathy Secondary to Cancer

The association of increased malignancy risk with glomerulonephritis is well-known (45–47), Lefaucheur et al. (48) found that 24 of 240 patients with MN developed a malignancy at the time of renal biopsy or within a year later. Compared with the general population, the incidence of cancer was 9.8 times higher for men and 12.3 higher for women, independently of age. Patients with MN and cancer were more frequently heavy smoker than controls. The risk of cancer associated MN increased with age, being around 2% in patients with <55 years and reaching 20–25% after 60 years. In a Norwegian study based on registry data of both cancer and MN, cancer was present at time of diagnosis of MN in 11 out of 166 patients with MN, and it was diagnosed in a median time of 60 months later in other 24 patients (49). A systematic review and meta-analysis of 6 observational studies that included 785 MN patients reported that the prevalence of cancer in patients with MN was 10%. The mean age of patients with cancer was 67 years and 2/3 of patients were males. In 20% of patients, cancer was diagnosed before the development of MN, while the other cases were diagnosed at time of renal biopsy or during the follow-up (50). The message from these studies is that the search for malignancy is warranted in patients with MN over the age of 55–60 years (51).

Which criteria should be used to define the causal relationship between MN and malignancy? This remains an open problem. The simultaneous or close diagnosis of both MN and cancer, the remission of proteinuria in cases of healing of neoplasia and its recurrence if neoplasia recurs should be the best criteria for defining this association and for minimizing detection bias. However, many patients with malignancies and proteinuria are not submitted to renal biopsy in consideration of the limited possibility of treating renal diseases. On the other hand, the risk of development of cancer may persist for a long time. In a study, the mean annual incidence ratio of cancer was 2.1/100 person-years in the 0–5-year period and 2.8/100 person-years for the 5–15 years after kidney biopsy (49).

**Figure 2:**
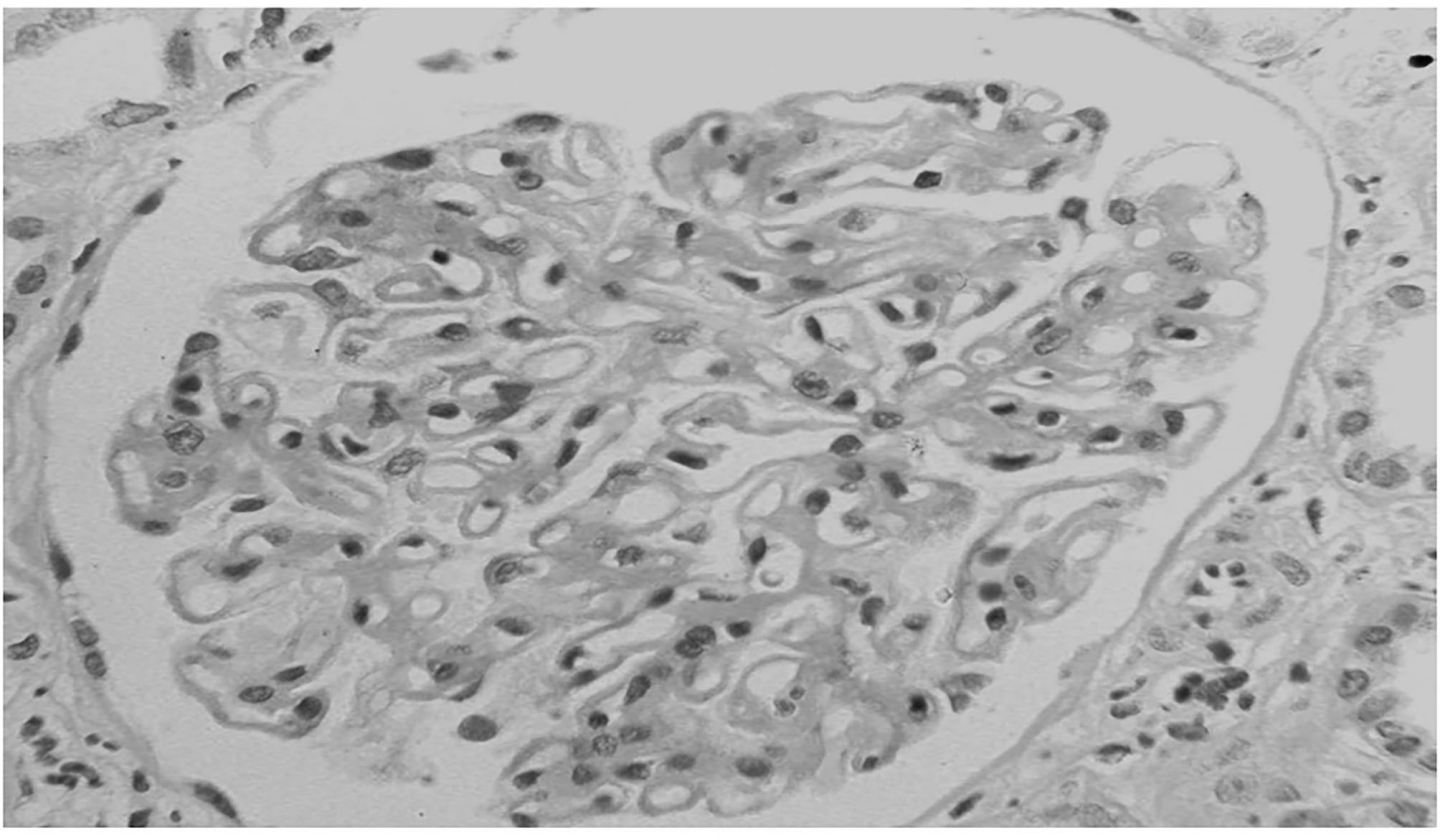
Membranous nephropathy in a patient with lung cancer. At light microscopy together with the diffuse thickening of glomerular capillary walls, some infiltration of leukocytes is present in capillary lumens.

Proteinuria is the main clinical manifestation and can be associated with renal failure and arterial hypertension in several cases. Although the histological presentation is similar to that of primary MN, some characteristics may identify the forms associated to cancer. At renal biopsy, glomeruli may be almost normal at optic microscopy, but mesangial hypercellularity together with infiltration of leukocytes in glomerular capillaries lumen have been reported in cases with underlying neoplasia. In particular, the presence of at least eight inflammatory cells per glomerulus was able to identify the forms associated with cancer with a specificity of 75% and a sensitivity of 92% in the study of Lefaucheur et al. (48) (Figure 2). At immunofluorescence subepithelial deposits of IgG1 and IgG2 are frequently detected in cancer associated MN while IgG4 dominates in idiopathic MN (52). This difference may represent one of the criteria for the differential diagnosis with primary MN (53) (Table 1). Compared with PLA2R- and THSD7A-positive forms of MN, there was a greater proportion of cases with malignancies in the nerve epidermal growth factor-like 1(NELL1)-associated group. Thus, NELL1-associated MN has a unique histopathology characterized by incomplete capillary loop staining, IgG1-predominance, and is more often associated with malignancy than other known types of MN (54).

Rare cases of detection of tumor antigens in subepithelial immune deposits of GBM together with antitumor antibodies have been reported (55). Beck et al. (56) proposed different mechanistic interpretations to explain the role of cancer in the development of MN; they include (i) the expression of planted tumor antigens triggering the production of circulation antibodies and the *in situ* formation of immune complexes composed by tumor antigens and antibodies; (ii) the formation of antibodies against a tumor antigen immunologically similar to a podocyte antigen; (iii) an abnormal immune response activated by an extrinsic process such as a viral infection. However, these hypotheses have not been confirmed because of the lack of a reliable experimental model. A simultaneous expression of THSD7A in gallbladder carcinoma and kidney has been described in a woman with MN. Moreover, out of 25 patients with MN and circulating anti- THSD7A antibodies, 7 had a malignant tumor (57). It has been outlined that patients with anti-PLA2R-negative MN are at higher risk of having a cancer-related form of MN compared to patients with anti-PLA2R positive MN (58). However, in a study, anti-PLA2R antibodies have been detected in 3 out of 10 patients with solid tumors. In these 3 patients there was a moderate subepithelial deposition of IgG4 (59). The cancers most frequently associated with MN are solid tumors such as lung, gastrointestinal, and prostate and uterus carcinoma, although cases associated with hematologic neoplasia and less frequently to melanoma have been reported (60, 61).

In view of the potential risks of underestimating a possible diagnosis of malignancy associated with MN a work-up to search for malignancies is suggested not only at the time of diagnosis of MN, but also during the follow-up in patients with negative anti-PLA2R1 antibodies and after the exclusion of other secondary forms of MN. The presence of anemia may also raise the suspicion of malignancy. Particularly in old patients, the work-up should include colonoscopy, prostate specific antigen search, mammography, and chest imaging in smokers (62). Once the diagnosis is done, the treatment should be directed to the associated cancer (63, 64). For patients with nephrotic syndrome, symptomatic treatment with ACE inhibitors and diuretics is indicated. Prevention of thromboses with anticoagulation in severe nephrotic syndrome is suggested. As expected, the prognosis of patients with cancer associated MN is worse than that of idiopathic forms.

## Hematopoietic Stem Cell Transplant (HSCT)

MN is the most frequent glomerular diseases associated with HSCT. In a study, out of 14 patients who developed a nephrotic syndrome after HSCT, 10 had a biopsy-proven MN (65). Clinical presentation is characterized by proteinuria usually in a nephrotic range that develops months after HSCT and is often associated with graft vs. host disease (GVHD).

The pathogenesis of MN is still incompletely elucidated. Some investigators feel that MN may represent the renal manifestation of GVHD (66). Other data support an autoimmune hypothesis (67). T cells are key players in GVHD (68, 69), but B cells can also contribute through both antibody-dependent and antibody-independent mechanisms (70), suggesting that GVHD may trigger an autoimmune response. Indeed, development of autoantibodies in association with a chronic GVHD has been reported (71). These data and the absence of PLA2R antibodies (72) support the hypothesis that HSCT-associated MN is a disease secondary to an autoimmune reaction to an allogeneic transplant (73).

Treatment depends on the amount of proteinuria. ACE inhibitors may be used in case of asymptomatic proteinuria. Most patients with nephrotic syndrome can respond to glucocorticoids, cyclosporine, or rituximab (74–76). However, a few patients are refractory to therapy and progress to ESRD.

## Membranous Nephropathy Secondary to Immunological and Rheumatological Diseases

### Lupus Membranous Nephropathy (LMN)

**Figure 3:**
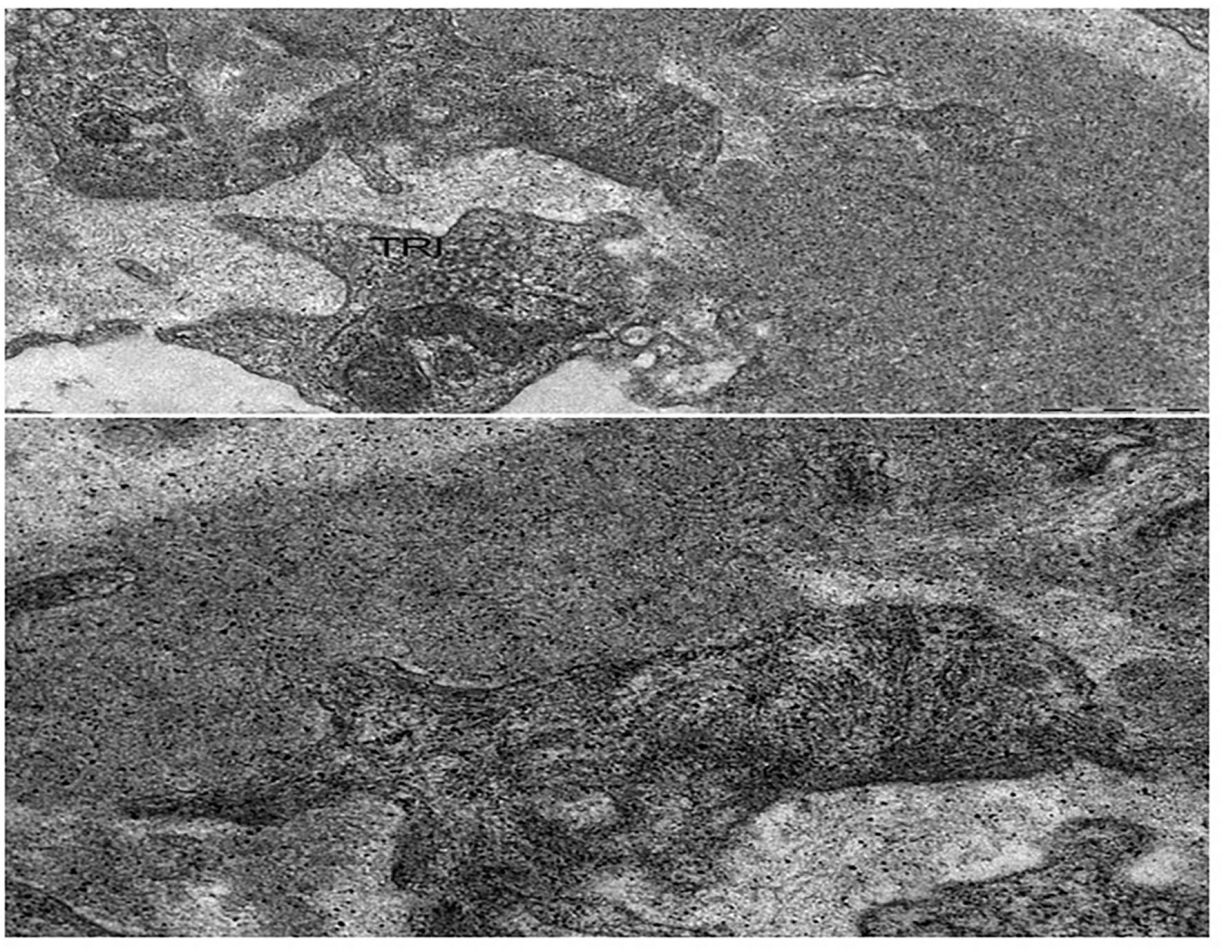
Lupus membranous nephropathy. At electron microscopy, sub-endothelial immune complexes deposits, structured in aggregates of concentric lamellae to form “finger prints” images; a tubulo-reticular inclusion (TRI) is observed in the endothelial cell. Bars: 500 nm.

LMN is a rare subtype of lupus nephritis. It accounts for ~15–20% of cases of lupus nephritis and mainly affects females (77). The mean age at presentation ranges around 30–35 years. In the current classification of lupus nephritis, MN is categorized as class V and includes cases of global or segmental subepithelial immune deposits with or without mesangial alterations (78). At light microscopy, LMN shares the similar characteristics of primary MN (Table 1). Mesangial proliferation is minimal or absent in primary MN while it may be present in LMN. At immunofluorescent study, the most important features in LMN are “full-house” deposits of IgG, IgM, IgA, and intense C1q and C4 staining in subepithelial and occasionally mesangial position. Deposits of IgG 1-3 may also distinguish lupus from primary MN, in which deposits of IgG4 are preponderant (79). The electron microscopy may show subepithelial and subendothelial deposits together with tubulo-reticular structures (“Interferon-fingerprints”) in endothelial cells (Figure 3). A recent study detected exostosin 1 (EXT1) and exostosin 2 (EXT2) staining in 80% of 26 patients with PLA2R negative MN and clinical features of autoimmune disease including lupus. Although serum EXT antibodies were not detected, these proteins may represent putative antigens in patients with this distinct subtype of secondary MN (80).

The pathogenesis of lupus MN is still incompletely elucidated. Some studies showed that insufficient apoptosis and neutrophil extracellular traps (NET) favor the exposure of nucleic acids and their binding proteins that are recognized as autoantigens (81–83). The loss of self-tolerance (84) favors the production of circulating antibodies that bind to autoantigen in podocyte membrane with consequent *in situ* formation of subepithelial immune deposits (85), which activate the lytic late components of complement (C5b-C9), release T cells and inflammatory cells, and produce reactive oxygen species. This sequence of events leads to glomerular injury and dysfunction of the glomerular barrier resulting in proteinuria.

The clinical presentation of LMN is variable. Some patients do not have extrarenal signs or symptoms of lupus and present with proteinuria and abnormal urinary sediment as the sole renal manifestations. In symptomatic patients, proteinuria may exceed 3.5 g per day and may be associated with hypoalbuminemia, dyslipidemia and variable degrees of edema. Hematuria and hypertension are common. Renal function is usually normal or subnormal.

Only few studies reported the long-term outcome of LMN. Progression to ESRD is slow and 72–97% of patients are still alive with kidney functioning at 10 years (86–90). Renal prognosis is largely influenced by the development of renal flares and transformation to proliferative disorders. Renal flares may be subdivided into proteinuric flares, characterized by increase in proteinuria with stable kidney function, and nephritic flares, characterized by a substantial increase in serum creatinine (91). Nephritic flares are difficult to manage and may lead to irreversible lesions, while proteinuric flares usually respond to treatment although remission may occur after weeks or months (92), Flares in patients with LMN are frequently associated with conversion to proliferative glomerulonephritis, as shown by repeat renal biopsy (93–95). Persistent nephrotic syndrome and prolonged use of corticosteroids can be responsible of dyslipidemia, diabetes, arterial hypertension, and hypercoagulability (96–98).

There is agreement that patients with persistent nephrotic syndrome despite the use of RAS inhibitors should receive immunosuppressive therapy, while there is controversy about the use of immunosuppression in patients with subnephrotic proteinuria. The Joint European League Against Rheumatism and European Renal Association-European Dialysis and Transplant Association (EULAR/ERA-EDTA) and several authorities recommended the use of corticosteroids and immunosuppressive drugs in pure class V nephritis, when the ratio urine protein/creatinine exceeds 1,000 mg/g despite the optimal use of renin–angiotensin–aldosterone system blockers (99–101). Corticosteroids alone are poorly effective, but they are largely used in combination with other immunosuppressive drugs. In a randomized controlled trial 42 patients were randomly assigned to prednisone alone or prednisone combined with cyclosporine for 11 months or alternate-month intravenous pulse cyclophosphamide for six doses. Both cyclophosphamide and cyclosporine were more effective than prednisone alone in inducing remissions of proteinuria, but relapses were more frequent after withdrawal with cyclosporine than cyclophosphamide (102). In the Aspreva trial (103) 370 patients with lupus nephritis were randomized to mycophenolate mofetil (MMF) or intravenous cyclophosphamide. In a pooled analysis of two randomized studies that included a subset of 65 participants with LMN, no difference was seen between response to MMF and to intravenous cyclophosphamide (104). Another randomized controlled study compared 6 months therapy of tacrolimus vs. MMF followed by azathioprine; all patients were also given high-dose prednisolone. The subgroup of patients with LMN treated with tacrolimus had significant more improvement of proteinuria and achieved more frequent complete and partial remission at 6 months in comparison to MMF (105). Years ago, we treated 11 LMN patients with a 6-month cyclical regimen based on alternating corticosteroids and cyclophosphamide every other month. After a mean follow-up of 83 months, 7 patients were in complete remission, and 3 patients were in partial remission (106). In the Rituxilup study, 22 patients with LMN were given 2 doses of rituximab (1 g) and methylprednisolone (500 mg) on days 1 and 15, and maintenance treatment with MMF. At 1 year, complete or partial remissions were achieved in >80% of patients (107). A systematic analysis of the use of rituximab in refractory lupus nephritis reported that this drug achieved complete or partial response in 67% of patients with refractory LMN (108). Thus, different treatments proved to be effective, but the choice depends on personal experience and previous treatments.

Whatever is the chosen immunosuppression, it is important not to neglect the treatment of complications, including hypertension, dyslipidemia, diabetes, thrombotic events, infections, and osteoskeletal disease.

### Rheumatoid Arthritis (RA)

As pointed out above, MN in RA was mainly secondary to the use of gold salts, penicillamine, or bucillamine. Today, these drugs are rarely used, and the frequency of MN decreased (109, 110). The lesions of RA-related MN are similar those of primary MN on examination by light microscopy, electron microscopy, and immunofluorescence (111). The pathogenetic mechanisms of RA-related MN are unclear. One may speculate that circulating autoantibodies in RA may target some podocyte proteins and eventually cause MN. Rituximab may probably represent the elective treatment of RA-related MN, being active both on RA and MN (112, 113).

### Urticarial Vasculitis (UV)

UV is an urticarial eruption that is often painful or has a burning sensation. The skin lesions consist of inflamed and reddened patches or weals that can persist of more than 24 h. There are two variants of UV: the normocomplementemic UV with a less severe clinical course, and the hypocomplementemic form which is considered as an immune complex-mediated disorder characterized by low serum levels of C1q, C2, C3, and C4 and the presence of circulating anti-C1q antibodies (114, 115). The skin lesions result from a cutaneous leukocytoclastic vasculitis of small vessels that mainly involves the skin but can also extend to joints, eye, lungs, gastrointestinal tract, and other organs (115). Hypocomplementemic UV is frequently associated with SLE, RA, drug reactions, infections, or malignancy (116, 117).

Cases of MN associated with UV have been reported (118, 119). A systematic review outlined that the most frequent glomerular disease in UV was membranoproliferative glomerulonephritis, 35% of cases, followed by mesangioproliferative glomerulonephritis, 21%, and MN, 19% (120). As for other systemic vasculitis, renal involvement carries a poorer prognosis, but the outcome can be improved by aggressive immunosuppressive treatment. Biologic agents, including omalizumab, corticosteroids, cyclophosphamide, MMF, cyclosporine, and hydroxychloroquine proved to be effective for both skin and systemic symptoms (121).

### IgG4 Membranous Nephropathy

IgG4-related disease is a fibroinflammatory disorder that can involve nearly any organ, including the kidney. Tubulointerstitial nephritis is the most common renal manifestation, but MN may also occur (122). PLA2R antibodies are negative in IgG4-MN (123). In contrast to primary MGN, granular C1q deposits are sometimes prominent, and concurrent tubulointerstitial nephritis is often seen. Elevation of serum IgG4 often accompanies IgG4-related disease; however, it is not specific in reaching the diagnosis. The pathogenesis of IgG4-related disease is not clarified. It responds promptly to steroids, although there is a high relapse rate on discontinuation of immunosuppression. In the case of steroid resistance, rituximab represents the second-line treatment (124).

### Sarcoidosis

Renal manifestations of sarcoidosis are rare but may occur at any age including childhood (125). Granulomatous interstitial nephritis and glomerulonephritis can occur. Sarcoidosis-associated glomerulonephritis includes a variety of histological forms, the most frequent being MN (126). In rare cases, both MN and granulomatous interstitial nephritis can be seen at renal biopsy (127). Glomerular disease may appear before, simultaneously or after other manifestations of sarcoidosis. There may be a long latency period between the development of active sarcoidosis and glomerular involvement and inversely (128).

Whether the presence of MN in patients with sarcoidosis is a mere coincidence or is due to a causal relationship is unclear (129). Anti-PLA2R antibodies in serum or PLA2R antigen in biopsy can be detected in patients with sarcoidosis and MN. The high prevalence of PLA2R antigen in patients with MN associated with active sarcoidosis should suggest a causal link between the two diseases (130). On the other hand, primary MN is rare in children, and anti-PLA2R antibodies may also been detected in a few cases of MN secondary to cancer or hepatitis B.

Corticosteroids have been largely used in patients with associated MN and granulomatous interstitial nephritis. In these cases, the rapid administration of high-dose corticosteroids may prevent irreversible interstitial fibrosis and tubular atrophy. In case of isolated MN with asymptomatic proteinuria, RAS inhibitors are used. In the presence of nephrotic syndrome, the treatment is similar to that adopted for primary MN.

### Autoimmune Thyroiditis

Autoimmune thyroiditis (Hashimoto or Graves' disease) is caused by autoantibodies directed against thyroid proteins, such as thyroglobulin, thyroid peroxidase, or thyroid stimulating hormone receptor. It is often associated with asymptomatic proteinuria and sometimes nephrotic syndrome. Different renal diseases may be detected in those instances, MN representing one of the most frequent underlying glomerular disease (131). Only rarely, MN caused by autoimmune thyroiditis is associated with profound hypothyroidism. In many patients with Hashimoto disease the free thyroxine level may be normal while thyrotropic stimulating hormone (TSH) is elevated. At least initially, symptoms of hypothyroidism may be absent or mild; in addition, ~37% of patients with primary MN show a decrease in serum triiodothyronine (132), so that the diagnosis may be difficult and may be done with delay.

The mechanisms linking autoimmune thyroiditis and MN are still poorly defined. However, both in children (133) and adults (134) the development of MN is associated with deposition of immune complexes mediated by anti- thyroid-peroxidase antibodies, suggesting that renal disease may be caused by the production of autoantibodies against podocyte antigens. Immunofluorescence examination demonstrates bright granular staining of IgG along the GBM, corresponding to glomerular granular staining of thyroid-peroxidase while no thyroglobulin deposits are present. Electron microscopy shows subepithelial electron-dense deposits.

The treatment of Hashimoto thyroiditis is thyroid hormone replacement with levothyroxine sodium. It is often enough to obtain remission of proteinuria. In case of severe nephrotic syndrome or superimposed crescentic glomerulonephritis (135), prednisone, and oral cyclophosphamide may be used.

### Sjögren Syndrome

Renal involvement is rare in Sjogren syndrome, affecting <10% of patients (136). Tubulointerstitial nephritis and tubular acidosis are prevalent but different glomerular diseases can also be seen (137, 138). A Chinese review of patients with Sjögren syndrome and biopsy-proven renal diseases reported that 36% had a MN (139). Light microscopy, immunofluorescence, and electron microscopy are similar to primary MN, but interstitial infiltrates are often present in Sjogren syndrome. The prognosis appears to be worse in patients with glomerular involvement, with lower survival rates and higher incidence of lymphoma compared to patients with predominantly tubulointerstitial involvement (140, 141). Transformations from MN to membranoproliferative glomerulonephritis or crescentic glomerulonephritis have been observed (142, 143). Little information is available about the effectiveness of corticosteroids or other immunosuppressive agents to slow progression of renal disease.

### Systemic Sclerosis

Renal involvement is common in systemic sclerosis. Some individuals are initially asymptomatic or show only mild proteinuria, microscopic haematuria, and occasional casts. These patients may follow an indolent course until hypertension and progressive deterioration of kidney function develop (144–146). Cases of MN have been reported. In most cases they were related to the use of D-penicillamine (147), but in a few patients no cause but scleroderma was identified (148, 149). Subepithelial deposits were seen on electron microscopy, suggesting that autoantibodies directed against S-cl70, centromere or polymerase III (150) may cause formation *in situ* of immune complexes.

### Ankylosing Spondylitis

Amyloidosis is the most frequent glomerular disease in ankylosing spondylitis, but exceptional cases of MN have also been reported (151). Apart from few cases secondary to treatment with gold salts, at light microscopy, immunofluorescence, and electron microscopy the findings are similar to those of primary MN, but the negative PLA_2_R may suggest a diagnosis of secondary MN (152, 153). This would be confirmed by resolution of pain and rapid decrease in proteinuria after administration of adalimumab (153).

## Data Availability Statement

The raw data supporting the conclusions of this article will be made available by the authors, without undue reservation.

## Author Contributions

CP conceived the study. CP and GM contributed in reviewing the literature and in writing the paper. All authors contributed to the article and approved the submitted version.

## Conflict of Interest

The authors declare that the research was conducted in the absence of any commercial or financial relationships that could be construed as a potential conflict of interest.
